# Ephrin Receptors (Eph): EphA1, EphA5, and EphA7 Expression in Uveal Melanoma—Associations with Clinical Parameters and Patient Survival

**DOI:** 10.3390/life10100225

**Published:** 2020-09-30

**Authors:** Malgorzata Gajdzis, Stamatios Theocharis, Pawel Gajdzis, Nathalie Cassoux, Sophie Gardrat, Piotr Donizy, Jerzy Klijanienko, Radoslaw Kaczmarek

**Affiliations:** 1 Department of Ophthalmology, Wroclaw Medical University, 50-556 Wroclaw, Poland; gosiagajdzis@gmail.com (M.G.); radoslaw.kaczmarek@umed.wroc.pl (R.K.); 2 First Department of Pathology, National and Kapodistrian University of Athens, 15772 Athens, Greece; theocharis@ath.forthnet.gr; 3 Department of Pathology, Curie Institute, 75005 Paris, France; jerzy.klijanienko@curie.fr; 4 Department of Pathomorphology and Oncological Cytology, Wroclaw Medical University, 50-556 Wroclaw, Poland; piotrdonizy@wp.pl; 5 Department of Ophthalmology, Institut Curie, 75005 Paris, France; nathalie.cassoux@curie.net; 6 Department of Biopathology, Institut Curie, PSL Research University, 75005 Paris, France; sophie.gardrat@curie.net

**Keywords:** ephrin receptor, Eph, uveal melanoma, melanoma

## Abstract

Uveal melanoma is the most common primary intraocular malignancy in adults. The development of distant metastases is associated with a poor prognosis. Ephrine receptors (Eph) are the largest subpopulation of tyrosine kinase receptors. They play an important role in processes related to the formation and progression of cancer. The aim of the study was to evaluate the expression of ephrin receptors EphA1, EphA5, and EphA7 in uveal melanoma and its associations with clinicopathological parameters, overall survival, and disease-free survival. The study included 94 previously untreated patients who underwent enucleation due to uveal melanoma. High expression of EphA1 was positively correlated with a smaller tumor size, less frequent extra-scleral extension, lower mitotic activity, and more frequent vitreous hemorrhage. High expression of EphA5 was associated with less frequent chromosome 3 loss, absence of distant metastases, and more frequent vitreous hemorrhage. High expression of EphA7 was associated with a more frequent primary tumor location in the posterior pole. High EphA5 expression was associated with longer overall survival time. The above findings indicate that high expression of EphA1 and EphA5 can be considered a beneficial prognostic factor in uveal melanoma.

## 1. Introduction

Uveal melanoma is the most common primary intraocular malignancy in adults, with a mean age-adjusted incidence of 5.1 cases per million per year [1]. 5-year survival rates depend on the stage of disease [2,3]. Uveal melanoma has a high tendency to metastasize, which is associated with dramatically poor prognosis. Approximately 50% of patients develop metastasis, irrespective of the type of treatment used in primary disease [4]. Only 8% of patients with metastatic melanoma survive 2 years [2]. Moreover, a meta-analysis of 29 studies conducted in 1988–2015 showed that the average progression-free survival and overall survival did not change over the years, regardless of the treatment method used [5]. This demonstrates the need to search for new prognostic factors and new potential treatment options.

Transmembrane ephrin receptors (Eph) constitute the largest sub-family of receptor tyrosine kinases (RTK) [6,7]. They are divided into two sub-groups, based on their ligand-binding affinity and structure of the extracellular domain. Currently, nine EphA (EphA1-A8, EphA10) and five EphB (EphB1-B4, EphB6) receptors have been identified. The ligands for Eph receptors are ephrins—membrane-anchored proteins, also divided into two subclasses—Ephrin-A (A1-A6) and Ephrin-B (B1-B3) [8,9].

The unique feature of Eph receptors is the ability to bidirectional signaling [8]. It means the possibility of activating signal pathways both in cells expressing the receptor and in cells containing ligand. The effects of these reactions can be opposite [10]. Signaling leads to modification of the actin cytoskeleton and organization of microtubules through intracellular control proteins and the expression of surface adhesion molecules, thereby regulating tissue boundary formation, cell adhesion, migration, repulsion, and invasion ability [11]. Eph receptors and ephrins also play an important role in angiogenesis—a key process for tumorigenesis [8,12]. Moreover, Eph receptors mediate cell-to-cell interactions not only in tumor cells, but also in tumor microenvironment—stroma and vasculature, which make them an attractive target for new drug development [13].

Accumulative clinical evidence has demonstrated that Eph receptors expression is associated with clinicopathological parameters important for patient management and prognosis in a variety of tumors [13,14,15,16,17,18]. Among others, there are reports about the role of Eph receptors in skin melanomas [19,20]. However, there is no comprehensive available data concerning the clinical significance of Eph receptors expression in uveal melanoma, whose biology is significantly different from skin melanomas. The present study aimed to assess EphA1, EphA5, and EphA7 expression in uveal melanoma, combined with clinicopathological parameters, overall survival, and disease-free survival.

## 2. Results

94 patients with choroidal melanoma were included in the study. High EphA1, EphA5, and EphA7 expression was noted in 26 (29.5%), 13 (14.3%), and 14 (15.6%) cases, respectively. A summary of the expression results is shown in Table 1. All Eph receptors present mainly cytoplasmic and occasionally membranous pattern of staining.

The total high expression of EphA1 (Table 2) was statistically associated with smaller tumor size (*p* = 0.048), less frequently occurring extra-scleral infiltration (*p* = 0.030), lower mitotic activity (*p* = 0.042), and more frequent presence of hemorrhage in the vitreous chamber (*p* = 0.014). Chromosome 3 loss was statistically associated with less frequent when reaction intensity was high (*p* = 0.001), but high total EphA1 expression showed only a trend of correlation with rarer chromosome 3 loss (*p* = 0.064). Moreover, high reaction intensity showed significant associations with absence of distant metastases (*p* = 0.045).

Total high EphA5 expression (Table 3) was associated with less frequent loss of chromosome 3 (*p* < 0.001), absence of distant metastases (*p* = 0.010), and more frequent occurrence of vitreous hemorrhage (*p* = 0.013). A trend of correlation between total EphA5 expression and lower mitotic activity was observed (*p* = 0.075), but high reaction intensity was statistically significant associated with lower mitotic activity (*p* = 0.023).

In the case of EphA7, the fewest associations between expression and clinical–pathological parameters were observed. Analyzing the intensity of the reaction, it was only found that distant metastases were less frequent in high intensity cases (*p* = 0.031). However, more frequent localization in the posterior pole was associated with total high EphA7 expression (*p* = 0.043) (Table 4).

**Figure 1:**
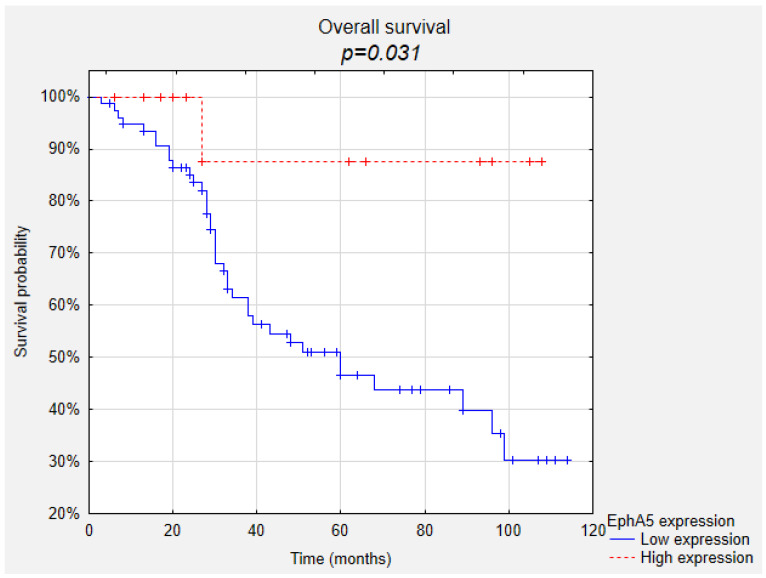
Correlation between EphA5 expression and overall survival.

**Figure 2:**
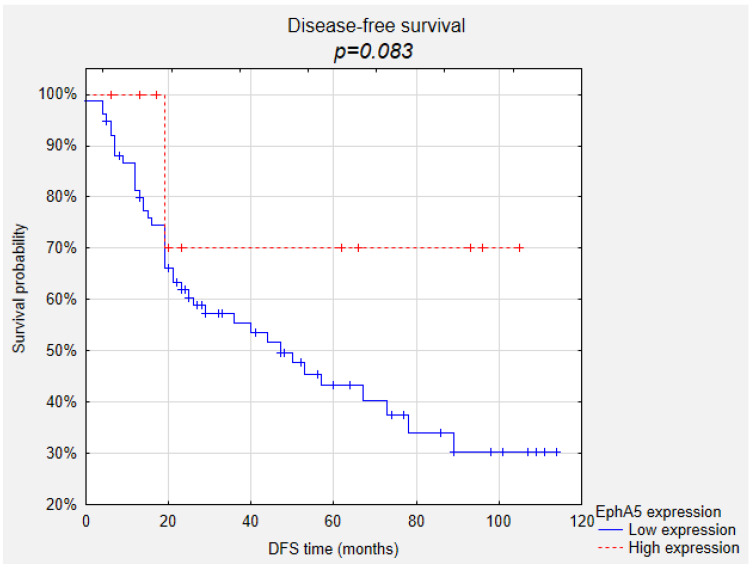
Correlation between EphA5 expression and disease-free survival.

Kaplan–Meier survival curves indicated that uveal melanoma patients with high EphA5 expression presented significantly longer overall survival periods compared to those presenting low EphA5 expression (Figure 1, *p* = 0.031). Also, high EphA5 expression showed a trend of correlation with higher probability of longer disease-free survival (Figure 2, *p* = 0.083). Both high reaction intensity and high percentage of positive cells tended to increase the likelihood of survival (*p* = 0.077 and *p* = 0.087, respectively). The high intensity of the reaction against EphA5 also increased the likelihood of longer disease-free survival (*p* = 0.038). EphA1 and EphA7 expression showed no correlations with overall survival and disease-free survival.

## 3. Discussion

Uveal melanoma, as the most common primary intraocular tumors in adults, is a significant problem in ophthalmic oncology. At diagnosis, less than 4% of patients have distant metastases, but eventually they occur in about half of the patients, often many years after primary treatment. This inevitably leads to death because there is no effective treatment for generalized disease. The search for new reliable prognostic factors is important because it allows the discovery of higher-risk patients.

Eph receptors, due to the wide prevalence in tissues, are a very interesting research object. In recent years, many reports have been published in which the expression of Eph receptors in various tumors has been assessed and its associations with clinical and histopathological parameters have been analyzed. It is worth emphasizing that the research results are divergent for each of the receptors analyzed. Depending on the cancer, both increased and decreased expression may be associated with a worse prognosis. This is because the effect of Eph/ephrin signaling on cellular processes is complex and highly dependent on the cellular context and stage of the disease [11]. Additional aspects are ligand-independent reactions, a change in signal strength depending on the receptors oligomerization, as well as the interaction of Eph with other RTK receptors.

In esophageal squamous cell carcinoma, elevated expression of EphA1 is associated with presence of lymph node metastasis and more advanced disease [17]. In clear-cell renal cell carcinoma, high expression of EphA1 is significantly associated with young age, sex, and higher histopathological grade [21]. In gastric cancer, high expression of EphA1 is associated with lower tumor histological differentiation, the presence of lymph node and distant metastasis. With increased expression, patients have shorter overall survival times as well as shorter disease-free survival times [22]. In gastric cancers, high expression of EphA1 is an independent prognostic factor [23].

On the other hand, in colorectal cancers, low EphA1 expression is associated with shorter survival times, lower histological differentiation, and the presence of lymph node metastasis [24]. In squamous cell carcinoma of the tongue, high expression of EphA1 is associated with poorer tumor vasculature and less frequent lymph node metastasis [13]. Analysis of the results of this study showed that in uveal melanomas, high expression of EphA1 is associated with lower mitotic activity, smaller tumor size, less frequent extra-scleral infiltration, and less frequent loss of chromosome 3. These parameters are well known risk factors for metastasis development.

In hepatocellular carcinoma, elevated EphA5 expression is associated with a higher grade of histological malignancy according to Edmondson and infiltration of blood vessels and bile ducts, suggesting that the receptor may affect cancer invasion and promote distant metastasis [25]. In pancreatic adenocarcinomas, patients with moderate or high EphA5 and EphA7 expression had shorter survival times compared to the low-expression group [14].

On the other hand, low EphA5 expression is associated with worse prognosis in ovarian serous carcinoma [26]. In clear-cell renal cell carcinoma, a decrease in EphA5 expression is observed compared to healthy renal tubular epithelial cells. Low expression is associated with a higher degree of histological malignancy according to Fuhrman [27]. Similar associations were observed by Giaginis et al., showing that patients with renal cancer achieve longer survival times if EphA5 expression remains high [15]. Low EphA5 expression is associated with a higher grade on the TNM Classification of Malignant Tumors and Gleason scale—two basic prognostic factors in prostate cancer [28].

In uveal melanoma, as with EphA1, high EphA5 expression is also a favorable prognostic factor. High expression has been shown to be associated with less frequent chromosome 3 loss, lower mitotic activity, and less frequent distant metastases. A tendency to lower histopathological grade was also observed in tumors with high EphA5 expression.

Decreased EphA7 expression occurs in colorectal cancers, but it has not been associated with any clinical parameters [29]. In gastric carcinoma, increased expression occurs in young patients and those with advanced tumors, but it has also not been shown to be associated with any other clinical parameters [30]. In esophageal squamous cell carcinoma, low expression of EphA7 results in more frequent lymph node metastases, poorer tumor differentiation, and a higher grade in TNM classification. Patients with low EphA7 expression have shorter survival times compared to the high expression group [16].

Li et al. and Theocharis et al., conducting research on squamous cell carcinoma of the tongue, showed that higher EphA7 expression is associated with longer survival times. If EphA7 expression is reduced, invasiveness and the tendency to distant metastasis increase [13,18]. Based on their research, Giaginis et al. showed that EphA7 expression is an independent prognostic factor. They also found associations between EphA7 expression and patient age, the presence of fibrosis, and tumor size [15]. In turn, studies on glioblastoma have shown that patients with tumors expressing EphA7 have shorter survival times compared to the non-expressed group, and the degree of expression inversely correlates with survival [31].

This study also noticed associations between high intensity reaction against EphA7 and a lower risk of distant metastasis. However, this is the only parameter associated with the prognosis with which a statistically significant connection has been demonstrated, so it is difficult to draw any certain conclusions.

Of the receptors tested, only in the case of EphA5 was a statistically significant association demonstrated with the survival time and disease-free survival time. Higher expression means a longer predicted survival time, as well as a tendency for longer disease-free survival. These observations confirm the role of high EphA5 expression as a favorable prognostic factor.

The action of most drugs associated with Eph signaling pathways leads to inhibition of receptor activity. However, the results of this study indicate that in the case of uveal melanoma, stimulation seems more reasonable. The first tests carried out in laboratory conditions have shown that this is possible. Recombinant Eph extracellular fragments can act as both agonists and antagonists [32]. For example, the N-terminal extracellular fragment of EphA7, acting as an agonist, induced apoptosis in leukemia cells in xenotransplanted mice [33]. It has also been shown that EphA2 antibodies can activate the signaling pathways associated with this receptor [32]. Selected peptides also can activate Eph receptors [34].

The high expression of selected Ephs can be used as a gripping point for targeted therapy. Antibodies and peptides capable of binding to Eph can be used to transport drugs, toxins, or radioisotopes to cancer cells. This technology allows not only for treatment, but also for the imaging of selected cancers [34,35].

However, one limitation of our study should be noted—the relatively low number of cases with positive Eph expression (especially EphA5 and EphA7)—which may make it difficult to draw strong conclusions from the statistical analysis.

## 4. Materials and Methods

### 4.1. Patients

Medical records and archive histopathological specimens of 94 patients with uveal melanoma diagnosed in 2007–2008 at the Curie Institute, Paris, France were used in the study. All patients underwent enucleation as primary treatment. Patients with prior radiotherapy or chemotherapy were not included in the study. Clinical data was obtained from the medical documentation. The follow-up periods up to 115 months have been documented. The study was approved by the Bioethics Committee of the Wroclaw Medical University, Wroclaw, Poland.

In this study, clinical parameters that are well known prognostic factors in uveal melanoma were taken into account (age, tumor size, ciliary body involvement, intra- or extra-scleral extension, grading, mitotic activity, chromosome 3 loss, and presence of metastasis) as well as three additional parameters that most strongly affect the visual acuity—tumor location in the posterior pole, retinal detachment, and vitreous hemorrhage. Mitotic activity was assessed on X400 in 40 fields using hematoxylin and eosin staining and it was determined in 89 cases. In the remaining 5 cases, there was too much melanin in the tumor cells, preventing reliable evaluation. The histological grading was based on conventional criteria: G1—spindle cell melanoma (>90% spindle cells), G2—mixed cell melanoma (>10% epithelioid cells and <90% spindle cells), and G3—epithelioid cell melanoma (>90% epithelioid cells). Chromosome 3 loss analysis was available for 65 patients and it was assessed by CGH (comparative genomic hybridization), FISH (fluorescence in situ hybridization), or karyotype studies. Other data were obtained based on medical documentation analysis.

### 4.2. Immunohistochemistry

**Figure 3:**
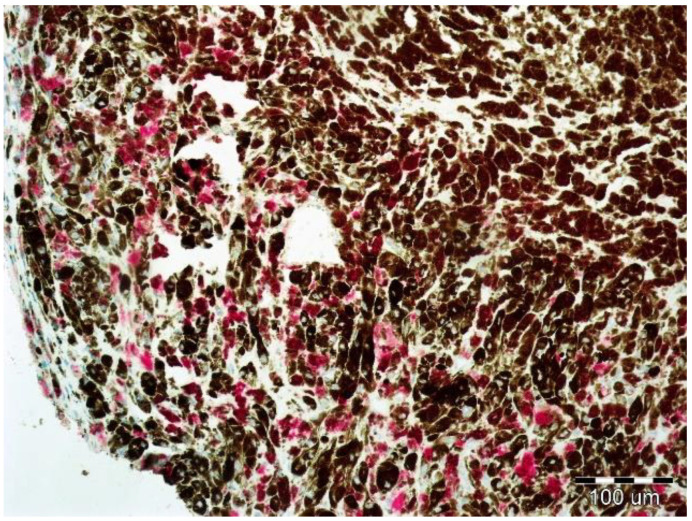
Uveal melanoma containing a large amount of melanin pigment. Positive reaction against EphA1.

Immunohistochemical staining to assess the expression of EphA1, EphA5, and EphA7 were performed using a red chromogen visualization kit enabling visualization in tissues containing a large amount of melanin (Figure 3). Commercially available rabbit polyclonal antibodies against EphA1 (ab5376, Abcam, Cambridge, United Kingdom), against EphA5 (ab5397, Abcam, Cambridge, UK), and against EphA7 (ab176102, Abcam, Cambridge, UK) were used for the study. As a positive control, hepatocellular carcinoma and breast cancer tissues were used, as recommended by the manufacturer.

**Figure 4:**
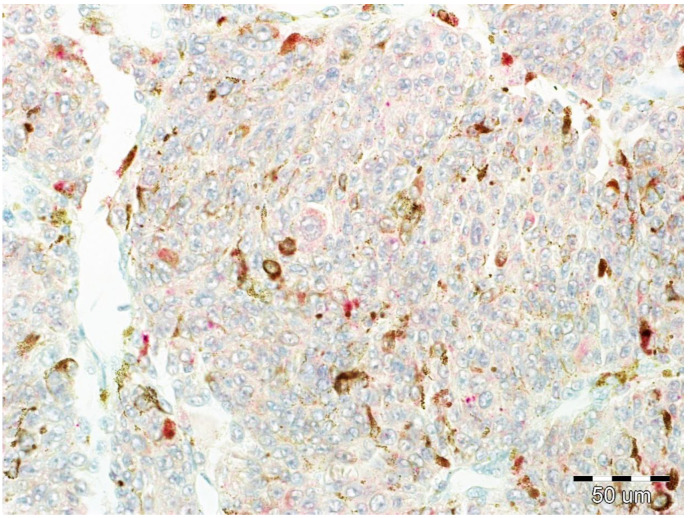
Example of low intensity cytoplasmic reaction against EphA5.

**Figure 5:**
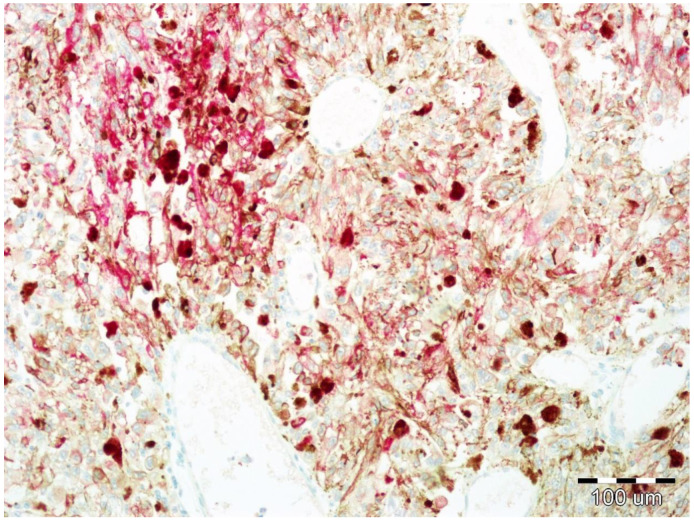
Example of moderate intensity cytoplasmic and membranous reaction against EphA1.

**Figure 6:**
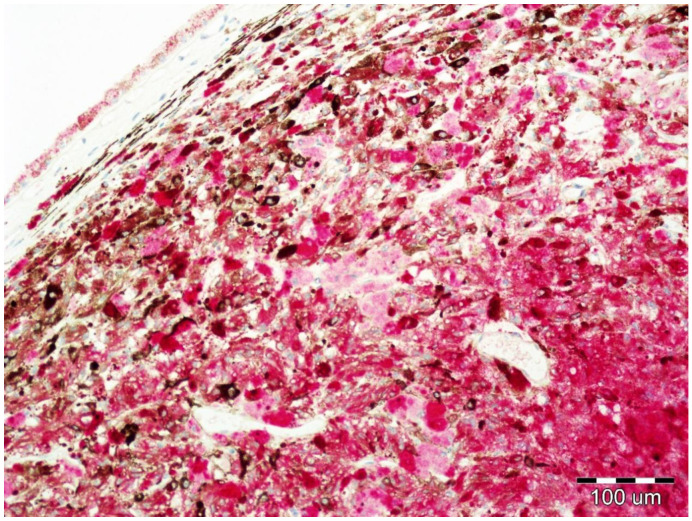
Example of a high intensity cytoplasmic reaction against EphA5.

Expression of EphA1, EphA5, and EphA7 was assessed by two independent pathologists (P.G. and S.T.) based on observation of at least 1000 cells in each case. To assess expression, the scale described in previous publications on the impact of Eph receptor expression on prognosis in other malignant neoplasms was used [13,14,15]. The immunoreactivity of the tumor cells was score according to the percentage of positive tumor cells as 0: 0–4% positive cells, negative staining; 1: 5–24% positive cells; 2: 25–49% positive cells; 3: 50–100% positive cells and reaction intensity scale 0: no reaction; 1: low reaction intensity (Figure 4); 2: moderate intensity (Figure 5); 3: high intensity (Figure 6). For the purposes of statistical analysis, 0–1 scores of positive tumor cells percentage were considered to be low, 2–3 as high, and similarly for reaction intensity: 0–1 scores were considered to be low reaction intensity and 2–3 as high reaction intensity. The total expression of EphA1, EphA5, and EphA7 was calculated based on the sum of the above two parameters (percentage of positive cells and intensity of the reaction) according to the “quickscore” method described for the assessment of estrogen receptor expression in breast cancer [36]. Cut off was set at ≥3 (low or no expression for a total of 0–2 and high expression for a total of 3 and more).

In the study group, the expression profile of EphA1, EphA5, and EphA7 receptors was examined in 88, 91, and 90 cases, respectively. 6, 3, and 4 cases, respectively, were removed from each group due to insufficient number of cancer cells in the examined tumor cross-section.

### 4.3. Statistical analysis

All statistical analyzes were carried out in the Statistica 13 program (StatSoft Polska, Krakow, Poland). Chi-square and U Mann–Whitney tests were used to assess the associations of EphA1, EphA5, and EphA7 protein expression and clinicopathological factors. Overall survival curves and disease-free survival curves were constructed using the Kaplan–Meier estimator. The differences between the curves were compared based on the log-rank test. The statistical significance limit was *p* < 0.05.

## 5. Conclusions

In the currently published English-language literature, there are no articles about the impact of Eph expression on the prognosis in uveal melanoma patients. Two non-English studies on the impact of EphA2 expression on prognosis have been published—one in German and one in Chinese [37,38]. The present study documented for the first time that some uveal melanomas express EphA1, EphA5, and EphA7 receptors. High expression of EphA1 and EphA5 can be considered a beneficial prognostic factor.

The above results indicate that it may be advisable to perform more frequent and more accurate tests for the presence of metastases (e.g., magnetic resonance imaging instead of liver ultrasound) in patients with low expression of EphA1 and EphA5 at the time of diagnosis. Moreover, the expression of EphA1, EphA5, and EphA7 in the examined tissues indicates them as a potential target for therapy.

The above results are an important starting point for further research on the role of Eph receptors in uveal melanomas. They can also be a new voice in the discussion about the pathogenesis and biology of this rare but potentially deadly disease. In addition, they can provide important information for further research into drugs effective in the treatment of uveal melanoma metastases, as well as drugs generally inhibiting the Eph/ephrin signaling pathway.

## Figures and Tables

**Table 1:** Distribution of EphA1, EphA5, and EphA7 expression in the examined tissues.

		EphA1	EphA5	EphA7
Reaction Intensity	Low	69 (7.4%)	79 (86.8%)	82 (91.1%)
	High	19 (21.6%)	12 (13.2%)	8 (8.9%)
Percentage of Positive Cells	Low	68 (77.3%)	83 (91.2%)	78 (86.7%)
	High	20 (22.7%)	8 (8.8%)	12 (13.3%)
Total Expression	Low	62 (70.5%)	78 (85.7%)	76 (84.4%)
	High	26 (29.5%)	13 (14.3%)	14 (15.6%)

**Table 2:** Associations of EphA1 expression with clinicopathological parameters in uveal melanoma patients.

Clinicopathological Parameters	EphA1 Low Expression (0–2)	EphA1 High Expression (3–6)	*p*-Value
**Age**			0.143
Mean 63, 63			
**Gender**			0.210
Male	28	8	
Female	34	18	
**Tumor Size**			0.048
≤9.0 mm	4	2	
9.1–12.0 mm	8	8	
12.1–15.0 mm	24	10	
>15.0 mm	26	6	
**Ciliary Body Involvement**			0.762
No	36	16	
Yes	26	10	
**Intrascleral Extension**			0.083
No	11	1	
Yes	51	25	
**Extra-Scleral Extension**			0.030
No	52	26	
Yes	10	0	
**Histopathological Grade**			0.690
G1	17	8	
G2	31	13	
G3	14	5	
**Mitotic Index/40 hpf**			0.042
0–5	40	21	
6–10	13	3	
>10	7	0	
**Chromosome 3 Loss**			0.064
No	8	6	
Yes	39	9	
**Metastases**			0.322
No	31	16	
Yes	31	10	
**Posterior Pole Involvement**			0.612
No	47	21	
Yes	15	5	
**Retinal Detachment**			0.487
No	36	13	
Yes	26	13	
**Vitreous Hemorrhage**			0.014
No	56	18	
Yes	6	8	

**Table 3:** Associations of EphA5 expression with clinicopathological parameters in uveal melanoma patients).

Clinicopathological Parameters	EphA5 Low Expression (0–2)	EphA5 High Expression (3–6)	*p*-Value
**Age**			0.683
Mean 64, 34			
**Gender**			0.163
Male	34	3	
Female	44	10	
**Tumor Size**			0.269
≤9.0 mm	5	2	
9.1–12.0 mm	14	2	
12.1–15.0 mm	28	6	
>15.0 mm	31	3	
**Ciliary Body Involvement**			0.341
No	43	9	
Yes	35	4	
**Intrascleral Extension**			0.463
No	12	1	
Yes	66	12	
**Extra-Scleral Extension**			0.171
No	68	13	
Yes	10	0	
**Histopathological Grade**			0.169
G1	19	7	
G2	42	3	
G3	17	3	
**Mitotic Index/40hpf**			0.075
0–5	50	12	
6–10	16	1	
>10	7	0	
**Chromosome 3 Loss**			<0.001
No	8	6	
Yes	47	3	
**Metastases**			0.010
No	36	11	
Yes	42	2	
**Posterior Pole Involvement**			0.121
No	63	8	
Yes	15	5	
**Retinal Detachment**			0.606
No	42	8	
Yes	36	5	
**Vitreous Hemorrhage**			0.013
No	69	8	
Yes	9	5	

**Table 4:** Associations of EphA7 expression with clinicopathological parameters in uveal melanoma patients.

Clinicopathological Parameters	EphA7 Low Expression (0–2)	EphA7 High Expression (3–6)	*p*-Value
**Age**			0.479
Mean 64, 18			
**Gender**			0.722
Male	31	5	
Female	45	9	
**Tumor Size**			0.425
≤9.0 mm	3	3	
9.1–12.0 mm	16	1	
12.1–15.0 mm	27	7	
>15.0 mm	30	3	
**Ciliary Body Involvement**			0.969
No	43	8	
Yes	33	6	
**Intrascleral Extension**			0.094
No	13	0	
Yes	63	14	
**Extra-Scleral Extension**			0.175
No	67	14	
Yes	9	0	
**Histopathological Grade**			0.366
G1	20	5	
G2	38	7	
G3	18	2	
**Mitotic Index/40 hpf**			1.000
0–5	50	11	
6–10	15	2	
>10	6	1	
**Chromosome 3 Loss**			0.744
No	11	2	
Yes	44	6	
**Metastases**			0.283
No	37	9	
Yes	39	5	
**Posterior Pole Involvement**			0.043
No	62	8	
Yes	14	6	
**Retinal Detachment**			0.649
No	43	7	
Yes	33	7	
**Vitreous hemorrhage**			0.509
No	65	11	
Yes	11	3	

## References

[1] Virgili G., Gatta G., Ciccolallo L., Capocaccia R., Biggeri A., Crocetti E., Lutz J.-M., Paci E. (2007). Incidence of Uveal Melanoma in Europe. Ophthalmology.

[2] Krantz B.A., Dave N., Komatsubara K.M., Marr B.P., Carvajal R.D. (2017). Uveal melanoma: Epidemiology, etiology, and treatment of primary disease. Clin. Ophthalmol..

[3] Kivela T., Simpson E.R., Grossniklaus H.E., Jager M.J., Singh A.D., Caminal J.M., Pavlick A.C., Kujala E., Coupland S.E., Finger P. (2017). Uveal melanoma. AJCC Cancer Staging Manual.

[4] Kujala E., Mäkitie T., Kivelä T. (2003). Very long-term prognosis of patients with malignant uveal melanoma. Investig. Ophthalmol. Vis. Sci..

[5] Yang J., Manson D.K., Marr B.P., Carvajal R.D. (2018). Treatment of uveal melanoma: Where are we now?. Ther. Adv. Med Oncol..

[6] Pasquale E.B. (2008). Eph-ephrin bidirectional signaling in physiology and disease. Cell.

[7] Himanen J.-P., Saha N., Nikolov D.B. (2007). Cell–cell signaling via Eph receptors and ephrins. Curr. Opin. Cell Biol..

[8] Surawska H., Ma P.C., Salgia R. (2004). The role of ephrins and Eph receptors in cancer. Cytokine Growth Factor Rev..

[9] Nievergall E., Lackmann M., Janes P.W. (2012). Eph-dependent cell-cell adhesion and segregation in development and cancer. Cell. Mol. Life Sci..

[10] Pasquale E.B. (2005). Eph receptor signalling casts a wide net on cell behaviour. Nat. Rev. Mol. Cell Biol..

[11] Tognolini M., Hassan-Mohamed I., Giorgio C., Zanotti I., Lodola A. (2014). Therapeutic perspectives of Eph–ephrin system modulation. Drug Discov. Today..

[12] Brantley D.M., Cheng N., Thompson E.J., Lin Q., Brekken R.A., E Thorpe P., Muraoka R.S., Cerretti D.P., Pozzi A., Jackson D. (2002). Soluble Eph A receptors inhibit tumor angiogenesis and progression in vivo. Oncogene.

[13] Theocharis S., Klijanienko J., Giaginis C., Alexandrou P., Patsouris E., Sastre-Garau X. (2014). Ephrin receptor (Eph) -A1, -A2, -A4 and -A7 expression in mobile tongue squamous cell carcinoma: Associations with clinicopathological parameters and patients survival. Pathol Oncol Res..

[14] Giaginis C., Tsourouflis G., Zizi-Serbetzoglou A., Kouraklis G., Chatzopoulou E., Dimakopoulou K., Theocharis S. (2010). Clinical significance of Ephrin (Eph)-A1, -A2, -A4, -A5 and -A7 receptors in pancreatic ductal adenocarcinoma. Pathol. Oncol. Res..

[15] Giaginis C., Tsoukalas N., Bournakis E., Alexandrou P., Kavantzas N., Patsouris E., Theocharis S. (2014). Ephrin (Eph) receptor A1, A4, A5 and A7 expression in human non-small cell lung carcinoma: Associations with clinicopathological parameters, tumor proliferative capacity and patients’ survival. BMC Clin. Pathol..

[16] Bai Y.-Q., Zhang J.-Y., Bai C.-Y., Xu X.-E., Wu J.-Y., Chen B., Wu Z.-Y., Wang S.-H., Shen J., Shen J.-H. (2015). Low EphA7 expression correlated with lymph node metastasis and poor prognosis of patients with esophageal squamous cell carcinoma. Acta Histochem. Cytochem..

[17] Wang J., Ma J., Dong Y., Shen Z., Ma H., Wang X., Shi S., Wu J., Lu G., Peng L. (2013). High expression of EphA1 in esophageal squamous cell carcinoma is associated with lymph node metastasis and advanced disease. APMIS.

[18] Li D., Xiang B., Ying X., Ying X., Dong H. (2014). Correlation analysis of EphA7 expression with clinico-pathological parameters and prognosis in tongue squamous cell carcinoma. Shanghai Kou Qiang Yi Xue.

[19] Hess A.R., Margaryan N.V., Seftor E.A., Hendrix M.J.C. (2007). Deciphering the signaling events that promote melanoma tumor cell vasculogenic mimicry and their link to embryonic vasculogenesis: Role of the Eph receptors. Dev. Dyn..

[20] Sakamoto A., Kato K., Hasegawa T., Ikeda S. (2018). An agonistic antibody to EPHA2 εxhibits antitumor effects on human melanoma cells. Anticancer Res..

[21] Wang X., Liu Y., Cao G., Zhang X., Xu H., Xu H., Wang J. (2015). Expression of the EphA1 protein is associated with Fuhrman nuclear grade in clear cell renal cell carcinomas. Int J. Clin. Exp. Pathol..

[22] Inokuchi M., Nakagawa M., Baogok N., Takagi Y., Tanioka T., Gokita K., Okuno K., Kojima K. (2018). Prognostic significance of high EphA1-4 expression levels in locally advanced gastric cancer. Anticancer Res..

[23] Nakagawa M., Inokuchi M., Takagi Y., Kato K., Sugita H., Otsuki S., Kojima K., Uetake H., Sugihara K. (2015). Erythropoietin-producing hepatocellular A1 is an independent prognostic factor for gastric cancer. Ann Surg. Oncol..

[24] Dong Y., Wang J., Sheng Z., Li G., Ma H., Wang X., Zhang R., Lu G., Hu Q., Sugimura H. (2009). Downregulation of EphA1 in colorectal carcinomas correlates with invasion and metastasis. Mod. Pathol..

[25] Wu J.-C., Sun B.-S., Ren N., Ye Q.-H., Qin L.-X. (2010). Genomic aberrations in hepatocellular carcinoma related to osteopontin expression detected by array-CGH. J. Cancer Res. Clin. Oncol..

[26] Chen X., Wang X., Wei X., Wang J. (2016). EphA5 protein, a potential marker for distinguishing histological grade and prognosis in ovarian serous carcinoma. J. Ovarian Res..

[27] Wang X., Xu H., Wu Z., Chen X., Wang J. (2017). Decreased expression of EphA5 is associated with Fuhrman nuclear grade and pathological tumour stage in ccRCC. Int. J. Exp. Pathol..

[28] Li S., Zhu Y., Ma C., Qiu Z., Zhang X., Kang Z., Wu Z., Wang H., Xu X., Zhang H. (2015). Downregulation of EphA5 by promoter methylation in human prostate cancer. BMC Cancer.

[29] Wang J., Kataoka H., Suzuki M., Sato N., Nakamura R., Tao H., Maruyama K., Isogaki J., Kanaoka S., Ihara M. (2005). Downregulation of EphA7 by hypermethylation in colorectal cancer. Oncogene.

[30] Wang J., Li G., Ma H., Bao Y., Wang X., Zhou H., Sheng Z., Sugimura H., Jin J., Zhou X. (2007). Differential expression of EphA7 receptor tyrosine kinase in gastric carcinoma. Hum. Pathol..

[31] Wang L.-F., Fokas E., Juricko J., You A., Rose F., Pagenstecher A., Engenhart-Cabillic R., An H.-X. (2008). Increased expression of EphA7 correlates with adverse outcome in primary and recurrent glioblastoma multiforme patients. BMC Cancer.

[32] Barquilla A., Pasquale E.B. (2015). Eph receptors and ephrins: Therapeutic opportunities. Annu. Rev. Pharmacol. Toxicol..

[33] Oricchio E., Nanjangud G., Wolfe A.L., Schatz J.H., Mavrakis K.J., Jiang M., Liu X., Bruno J., Heguy A., Olshen A.B. (2011). The Eph-receptor A7 is a soluble tumor suppressor for follicular lymphoma. Cell.

[34] Wang S., Placzek W.J., Stebbins J.L., Mitra S., Noberini R., Koolpe M., Zhang Z., Dahl R., Pasquale E.B., Pellecchia M. (2012). Novel targeted system to deliver chemotherapeutic drugs to EphA2-expressing cancer cells. J. Med. Chem..

[35] Cai W., Ebrahimnejad A., Chen K., Cao Q., Li Z., Tice D.A., Chen X. (2007). Quantitative radioimmunoPET imaging of EphA2 in tumor-bearing mice. Eur. J. Nucl. Med. Mol. Imaging.

[36] Detre S., Saclani Jotti G., Dowsett M. (1995). A “quickscore” method for immunohistochemical semiquantitation: Validation for oestrogen receptor in breast carcinomas. J. Clin. Pathol..

[37] Vukoja V., Brandenbusch T., Tura A., Nassar K., Rohrbach D.J.M., Luke M., Grisanti S. (2016). Expression of EphA2 in metastatic and non-metastatic primary uveal melanoma. Klin. Monbl. Augenheilkd..

[38] Chen L.-X., Sun B.-C., Li X., He Y.-J., Song G.-X. (2012). Overexpression of the receptor tyrosine kinase EphA2 in choroidal melanoma: Correlation with vesculogenic mimicry and prognosis. Chin. J. Ophthalmol..

